# The Elbs and Boyland-Sims peroxydisulfate oxidations

**DOI:** 10.1186/1860-5397-2-22

**Published:** 2006-11-07

**Authors:** E J Behrman

**Affiliations:** 1 Department of Biochemistry, The Ohio State University, 484 West 12th Avenue, Columbus, OH 43210, USA

## Abstract

This paper reviews the recent literature on the title reactions and updates a 1988 review.

The Elbs and Boyland-Sims oxidations are shown in Scheme 1:

**Scheme 1:**
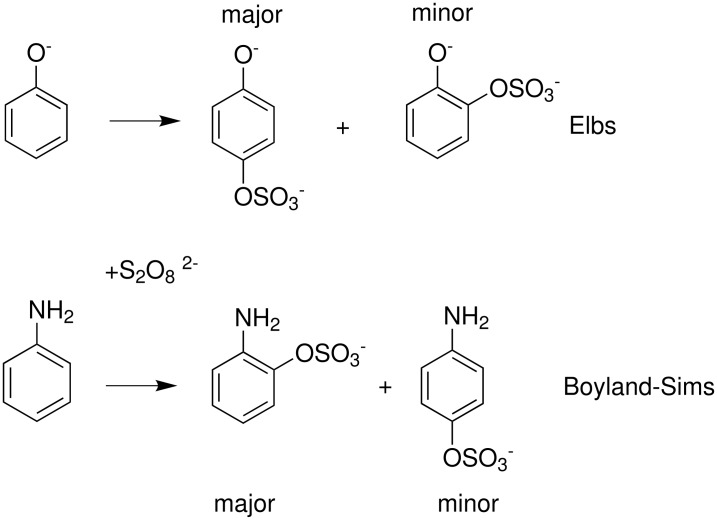
The Elbs and Boyland-Sims Oxidations.

Both of these reactions, last reviewed in 1988 [1], are nucleophilic displacements on a peroxide oxygen of the peroxydisulfate ion. In the Elbs oxidation, the nucleophile is a phenolate anion (or a tautomer) and in the Boyland-Sims oxidation, it is a neutral aromatic amine. There is no radical involvement in either case (except in side reactions, see below). The products are aromatic sulfates whose orientation relative to the phenolic group is preferentially *para* in the Elbs oxidation and *ortho* in the Boyland-Sims case. These sulfates are useful in synthesis themselves or may be hydrolyzed in acid to the dihydric phenols (or aminophenols).

The yields of products are typically low to moderate, but the simplicity of the reactions frequently recommends their use. Reactions are usually carried out by dissolving the phenol or amine in an alkaline aqueous medium, sometimes with the addition of a co-solvent such as pyridine, and then adding a peroxydisulfate salt. The ammonium and sodium salts are much more soluble than the potassium salt. This low solubility may be used to advantage to ensure slow addition of the peroxydisulfate as it is established that higher yields are achieved when the phenol(amine)-peroxydisulfate ratio is large. Isolation of the product usually takes advantage of the high water solubility of the intermediate sulfate ester; acidification of the reaction mixture is followed by extraction of the unreacted starting material (in the case of phenols) by an appropriate organic solvent. The sulfate ester remains in the aqueous phase. Hydrolysis of the sulfate ester in aqueous acid produces the (usually) organic-soluble dihydric phenol. Reactions are usually run at room temperature or below to reduce the incursion of free radical reactions. The rates are rather slow with typical second-order rate constants (at room temperature) in the range 0.1–20 L mol^-1^ min^-1^. [1]

## Mechanisms of the reactions

**Scheme 2:**
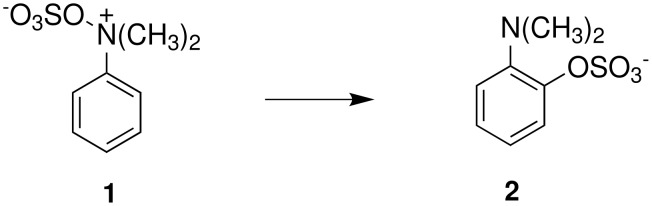
The Intermediate in the Boyland-Sims Oxidation

The mechanisms of both reactions have been clarified. In both cases this was done by synthesis of postulated intermediates. In the Boyland-Sims oxidation, the amine-O-sulfonate was the obvious choice that was supported by a number of kinetic studies. [1] This was questioned by Edward and Whiting [2] who claimed that the molecule formed by sulfonating *N*,*N*-dimethylaniline-*N*-oxide(**1**) did not rearrange to the *o*-sulfate(**2**) but rather decomposed by hydrolysis. However, repetition of this work under strictly anhydrous conditions showed that the sulfonated *N*-oxide(**1**) indeed rearranged to the *o*-sulfate(**2**) in good yield (Scheme 2). [3]

**Scheme 3:**
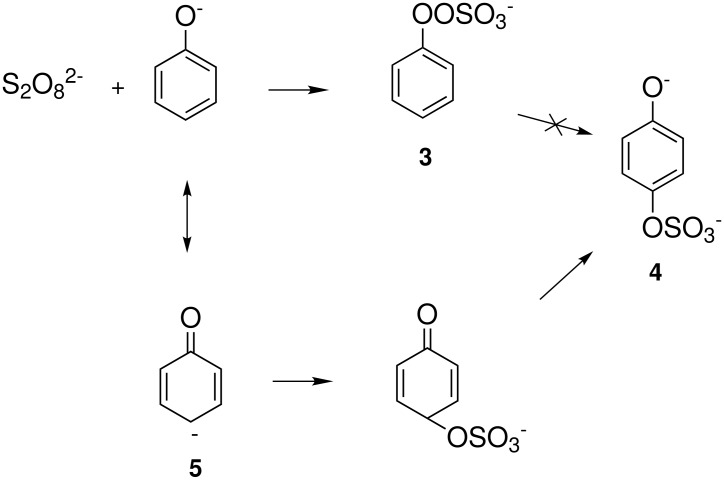
The Intermediate in the Elbs Oxidation.

In the Elbs oxidation, since the product is preferentially the *p*-sulfate, the question was whether the initial attack was at the phenolate oxygen(**3**) followed by rearrangement to the *p*-sulfate(**4**) or whether the initial attack was by the tautomeric *p*-carbanion(**5**) (Scheme 3).

**Scheme 4:**
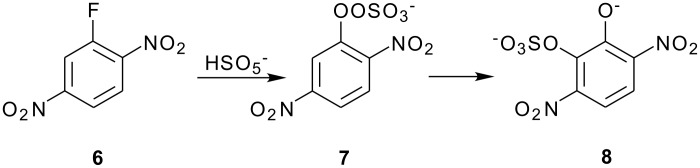
Reaction of Caro's Acid Anion with 2,5-dinitrofluorobenzene.

Here it was possible to synthesize the first possibility by displacement of the fluoride ion from three dinitrofluorobenzenes by reaction with Caro's acid anion (HSO_5_^-^). [4] The key example was for 2,5-dinitrofluorobenzene(**6**) which offers both an *ortho* and a *para* position for the rearrangement. Fluoride **6** reacted with Caro's acid anion to yield the intermediate **7**. Intermediate **7** rearranged strictly to the ortho isomer, **8**. This finding strongly supports attack in the Elbs oxidation by the *para*-carbanion tautomer of the phenolate anion, **5**, rather than through the intermediate **3** (Scheme 4).

This experimental finding was confirmed and expanded by calculations of the energies of the two pathways for phenol itself.

## Other recent developments

Among recent developments is the observation that the synthesis of 5-hydroxyorotic acid from orotic acid is markedly affected by oxygen [5] but in a way opposite to the more usual observation that yields are improved by excluding oxygen. Here, in the absence of oxygen, yields are low and the pyrimidine ring undergoes cleavage to urea. Oxygen inhibits this side-reaction and so increases the yield of the expected Elbs product, orotic acid 5-sulfate. Quinolones are another interesting case: 4-quinolones give reasonable yields of the expected 3-substitution products whereas 2-quinolones do not. [6] An explanation has been suggested which concerns the stability of the key intermediate in each case. This finding may be connected with the broader issue of low yields in the Elbs oxidation; yields are typically below 50% and no satisfactory explanation has so far been put forward (see below).

Watson and Serban have introduced a selective method for hydrolysis of the intermediate sulfate esters using acetic acid which spares carboxylic esters. These authors also give a current example of the use of sulfate esters as a protecting group in synthesis [7] as do Bunnett and Jenvey. [8]

Parenthetically, there is a disputed report of the formation of quinones in low yield by peroxydisulfate oxidation of phenols under acid conditions. [9–10]

## Discussion of some unresolved problems

### 1. The *ortho-para* ratio

**Scheme 5:**
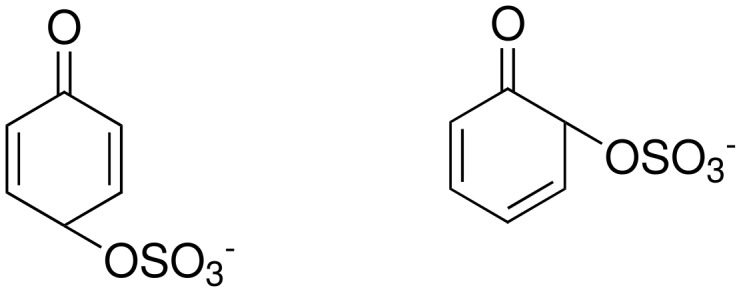
*Ortho*- and *para*-intermediates for the Elbs Oxidation.

We have previously calculated the energy difference between the *ortho-* and *para-* intermediates shown in Scheme 5; it is about 2 kcal mol^-1^. [4]

**Scheme 6:**
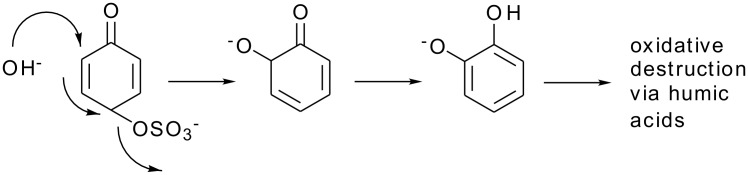
Reaction of an Elbs Intermediate with the Hydroxide Ion.

This calculation corresponds reasonably well, considering that it is for the gas phase, with the usual value of the *ortho-para* ratio in the products of an Elbs oxidation of about 0.1. [1] This ratio translates to an energy difference of 3 kcal mol^-1^. It is also striking that about the same energy difference exists between *o-* and *p*-benzoquinone where the difference in the redox potential is 0.07 V [11] corresponding to 3 kcal mol^-1^. It may also be relevant that quinones are subject to attack by the hydroxyl ion to form (eventually) humic acids and that *o*-quinones are more reactive than *p*-quinones. [12–13] An analogous reaction for an Elbs intermediate with the hydroxide ion is outlined in Scheme 6.

Reactions of this kind could account for part of the deleterious effects of excess alkali on yields in the Elbs oxidation [14] by formation of humic acids.

### 2. Low yields and recovery of starting material

**Scheme 7:**
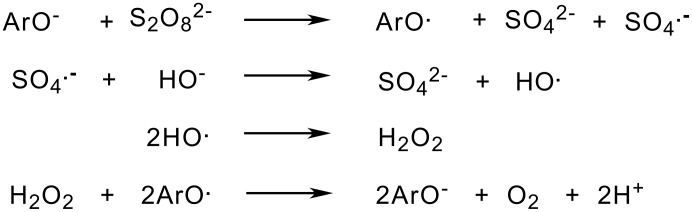
A Catalytic Cycle for Peroxydisulfate Consumption.

A significant disadvantage to the Elbs Oxidation is the low yield; yields are seldom above 50% But it is also characteristic that large quantities of starting material can be recovered. This is true even with a ratio of peroxydisulfate to phenol greater than one. The peroxydisulfate is always completely consumed. These facts imply a side-reaction in which the phenol serves as a catalyst in a set of reactions which leads to the consumption of peroxydisulfate. A particularly striking case is that of the 2-quinolones where no Elbs product is formed although peroxydisulfate is consumed at a reasonable rate. [6] *Para-*substituted phenols always give low yields in comparison with the *o*- and *m*-substituted isomers. Rao and Rao have found that increasing the peroxydisulfate-phenol ratio increases the yield for the *p*-isomers whereas for the *o*- and *m*-isomers the opposite is usually found. [15] The set of reactions shown in Scheme 7 is a possible explanation of this situation but fails to account for reality as no detectable oxygen is observed during the reaction between peroxydisulfate and either 2-quinolone or 2-quinolone-4-carboxylic acid. [16]

**Scheme 8:**
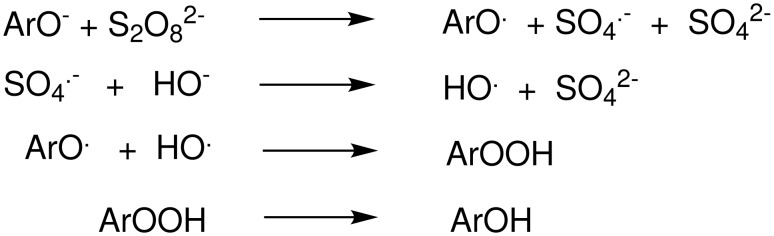
A Non-catalytic cycle for Peroxydisulfate Consumption.

An alternative explanation, not strictly catalytic, is shown in Scheme 8 which depends on the fact that aryl hydroperoxides are rather unstable and decompose rapidly to phenols unless electron-withdrawing elements are present in the structure. Note that the last equation in Scheme 8 is left unbalanced because the oxidized products of Elbs and Boyland-Sims reactions are poorly characterized. It seems clear that small proportions of starting materials, particularly for such substrates as the 2-quinolones, can undergo extensive oxidation probably involving catalysis by adventitious metal ions.

Thus Walling and Buckler [17] reacted phenyl magnesium bromide with oxygen and, although they obtained evidence for a peroxide, isolated only phenol (in substantial yield). Heller and Weiler [18] investigated a more stable analog, namely p-nitrophenyl hydroperoxide formed by *ipso* displacement of a nitro group from p-dinitrobenzene by the hydrogen peroxide anion.*p*-Nitrophenyl hydroperoxide forms *p*-nitrophenol on decomposition. Similarly, Malykhin and Shteingarts found naphthols as products from the reaction of potassium peroxide with several nitronaphthalenes. [19] These findings are then consistent with the hypothesis that aryl hydroperoxides formed by reaction of arylphenoxide radicals with the hydroxyl radical decompose to form the parent phenol in good yield with the proportion of reformed phenol to the Elbs product dependent on the phenol structure. Similar, but generally more stable aryl peroxides, ArOOR, have been prepared and undergo either decomposition to the phenol or, frequently, rearrangement. [4,20–22]

As this suggestion for the catalytic consumption of peroxydisulfate involves phenoxyl radicals, the relative stability, i. e. reactivity, of these radicals is important. There is some literature to suggest that substitution *ortho* or *meta* to the phenolic oxygen yields a more stable (less reactive) radical than the *para*-substituted case; Wright *et al*. have calculated bond dissociation energies for the formation of phenoxyl radicals from 36 *o-,m-*, and *p*-substituted phenols. [23] While there are a few exceptions (some irrelevant to this discussion), the *p*-substituted phenols generally have lower bond dissociation energies in the range of 1–5 kcal/mol than either the *o*- or *m*-substituted isomers in accord with this proposal. Bader and Jahngen [24] have reported a 9% increase in yield in the synthesis of gentisaldehyde in the presence of allylbenzene, a sulfate radical trap, although in other cases (such as *o*-nitrophenol [1]) radical traps have little effect. It seems clear that the relative rates of the radical and non-radical pathways vary widely with the nature of the phenol.

Other side-reactions include disulfation [25–26] and the formation of humic acid-like materials. [1] Also, displacement of substituents by *ipso* attack has been reported for R = -Cl [27], -I[28], -COOH[29], and -NO_2_ in the case of *p*-nitrophenol. [16]

## Future directions

Peroxydisulfate is a very versatile oxidant as recent reviews have pointed out. [30–31] An important developing area is the use of salts of peroxydisulfate which are soluble in organic solvents by virtue of large organic cations in place of the commercially available ammonium, sodium, or potassium salts. This approach has not yet been reported for the Elbs or Boyland-Sims oxidations.

Corresponding chemistry of the peroxyphosphates has been little developed[31–32] although there is a prelimary report of the use of peroxymonophosphate to carry out the synthesis of catechol monophosphates analgous to the reaction reported in [4] with peroxymonosulfate. [33]

Biographical material on Elbs (1858–1933) and Boyland (1905–2002) has appeared. [34–36]

The Tables record the literature on the Elbs and Boyland-Sims oxidations from 1984 (the approximate literature cutoff for ref. 1) through mid-2006. There are also a few earlier references which were omitted from ref. 1.

Key to the Tables: the Tables are grouped by compound type. Within each group, the starting materials are listed in order of increasing numbers of carbon atoms and then in order of increasing numbers of hydrogen atoms. Starting materials marked with an asterisk were not reported in ref. 1. Isolated yields are given in percent; if no yield was reported, that is indicated by a dash.

**Table 1:** Oxidation of phenols

**# of carbons**	**Phenol**	**Product(s) and yields (%)**	**Ref.**

6	2,3-difluorophenol*	2,3-difluorohydroquinone, 37	[37]
	2,6-difluorophenol*	2,6-difluorohydroquinone, 26	[37]
	"	", 22	[38]
	3,5-difluorophenol*	", ca. 20	[38]
	2,5-dinitrophenol*	2,5-dinitrohydroquinone, 36	[39]
	2,6-dinitrophenol*	2,6-dinitrohydroquinone, 35	[39]
	2-fluorophenol	2-fluorohydroquinone, 47	[37]
	2-nitrophenol	2-nitrohydroquinone, 35	[40]
	3-nitrophenol	2-nitrohydroquinone, 10; 2-nitro-4-hydroxyphenylsulfate, 13	[41]
	4-nitrophenol	2-hydroxy-5-nitrophenylsulfate, 5	[42]
	"	", -	[43]
	"	5-nitropyrogallol, 1–10	[25]
	phenol	4-hydroxyphenylsulfate, 46	[7]
	"	", -	[44]
7	2-methylphenol	4-methoxy-3-methylphenol, 1.4	[8]
	3-methylphenol	4-methoxy-2-methylphenol, 8	[8]
	"	2-methylhydroquinone, 66	[45]
8	2,6-dimethoxyphenol*	2,6-dimethoxyhydroquinone, 69	[45]
	2-hydroxy-3-methoxybenzaldehyde*	2,5-dihydroxy-3-methoxybenzaldehyde, -	[46]
	2,5-dichloro-3,6-dimethoxyphenol*	2,5-dichloro-3,6-dimethoxyhydroquinone, 42	[47]
	2,3-dimethylphenol*	2,3-dimethylhydroquinone, 20	[38]
	"	", 49	[48]
	2,6-dimethylphenol	2,6-dimethylhydroquinone, 35	[49]
	3,4-dimethylphenol*	3,4-dimethylcatechol, 16	[49]
	2,4-demethylphenol*	dimer, 2	[49]
9	2-hydroxy-6-methoxyacetophenone	2,5-dihydroxy-6-methoxyacetophenone, 3	[50]
	tyrosine*	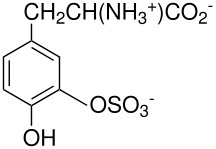 , 20	[51]
10	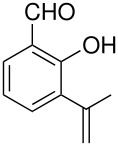 *	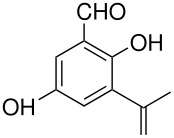 ,-	[52]
	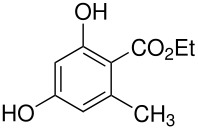 *	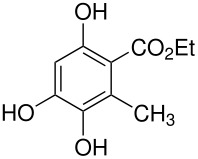 , 15	[53]
	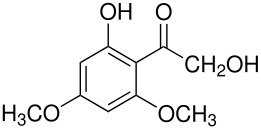 *	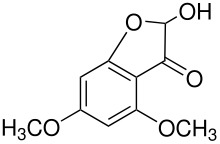 , -	[54]
11	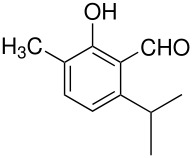 *	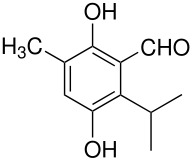 , 7	[52]
	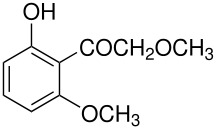	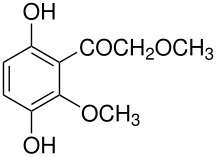 , 22	[55]
	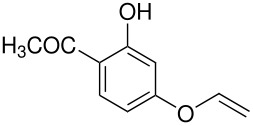 *	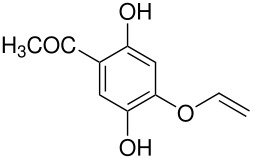 ,24	[56]
12	p-hydroxydiphenyl*	Failed	[57]
14	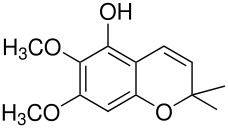 *	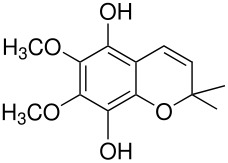 ,-	[58]
15	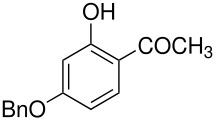	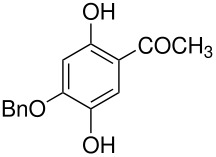 , 39	[55]
	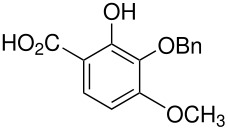 *	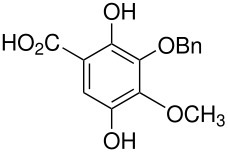 , 23	[59]
17	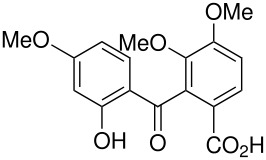 *	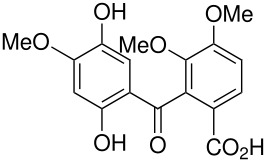 ,30	[60]
18	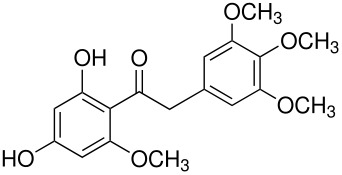 *	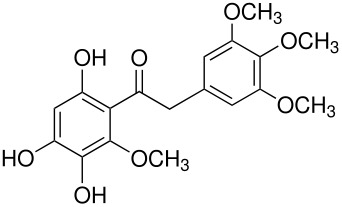 , 21	[61]
22	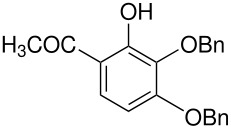 *	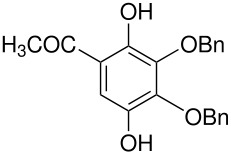 , 10	[62]

**Table 2:** Oxidation of anilines

**# of carbons**	**Anilines**	**Product(s) and yields (%)**	**Ref.**

6	3,4-dichloroaniline*	3,4-dichloroaniline-2-sulfate, 3.5; 3,4-dichloroaniline-6-sulfate, 2.5	[63]
7	3-fluoro-4-methylaniline* (the starting material is misnamed in the original)	3-fluoro-4-methylaniline 2- & 6-sulfate, -; & the corresponding phenols, -	[64]
12	2-aminodiphenyl	2-aminodiphenyl 3- & 5-sulfates,-	[65]
22	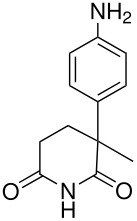 *	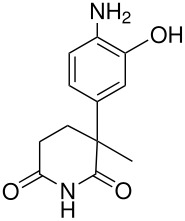 , 6	[66]

**Table 3:** Oxidation of coumarins

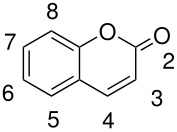			
**# of carbons**	**Coumarin**	**Product(s) and yields (%)**	**Ref.**

11	4-methyl-7-methoxycoumarin	4-methyl-7-methoxy-6-hydroxycoumarin, 10	[67]
	3-methyl-7-methoxycoumarin*	3-methyl-7-methoxy-6-hydroxycoumarin, 4	[68]
	3-methyl-8-methoxycoumarin*	3-methyl-8-methoxy-6-hydroxycoumarin, 7	[68]
12	4-methyl-5-hydroxy-6-acetoxycoumarin*	4-methyl-5,8-dihydroxy-6-acetoxycoumarin, 30	[67]
	4-methyl-5,7-dimethoxycoumarin	4-methyl-5,7-dimethoxy-6-hydroxycoumarin, 13	[67]
	4-methyl-7,8-dimethoxycoumarin	4-methyl-7,8-dimethoxy-6-hydroxycoumarin, 60	[67]
14	4-methyl-7-diethylaminocoumarin*	?	[69]
17	4-phenyl-5,7-dimethoxycoumarin	4-phenyl-5,7-dimethoxy-6-hydroxycoumarin, 6	[67]

**Table 4:** Oxidation of xanthones

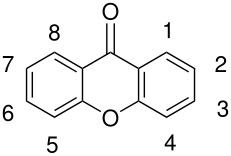			
**# of carbons**	**Xanthone**	**Product(s) and yields (%)**	**Ref.**

13	1,3,8-trihydroxyxanthone*	1,3,5,8-tetrahydroxyxanthone, 36	[70]
15	1-hydroxy-3,8-dimethoxyxanthone*	Failure	[71]

**Table 5:** Oxidation of flavones

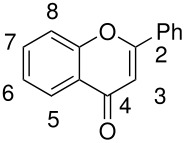			
**# of carbons**	**Flavone**	**Product(s) and yields (%)**	**Ref.**

15	5,7-dihydroxyflavone	5,7-dihydroxyflavone-8-sulfate, 43	[72]
22	5-hydroxy-7-benzyloxyflavone	5,8-dihydroxy-7-benzyloxyflavone, -	[73]

**Table 6:** Oxidation of pyridines

**# of carbons**	**Pyridine**	**Product(s) and yields (%)**	**Ref.**

5	2-pyridone	5-hydroxy-2-pyridone, -	[74]
	"	", -	[75]
	"	", 38	[76]
6	6-methyl-2-pyridone	5-hydroxy-6-methyl-2-pyridone,-	[75]

**Table 7:** Oxidation of pyrimidines

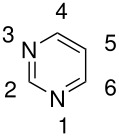			
**# of carbons**	**Pyrimidine**	**Product(s) and yields (%)**	**Ref.**

4	Uracil	5-hydroxyuracil, 50	[77]
	6-hydroxycytosine*	6-hydroxycytosine-5-sulfate, 60	[77]
5	orotic acid(uracil-6-carboxylic acid)*	Orotic acid-5-sulfate, 47; 5-hydroxyorotic acid, 46	[5]
	6-methyluracil	5-hydroxy-6-methyluracil, 50	[78]
	"	", 19	[79]
	6-methyluracil-2-^14^C*	5-hydroxy-6-methyluracil-2-^14^C, 20	[80]
	6-methyluracil	6-methyluracil-5-sulfate, 24	[79]
	6-methyluracil-4,6-^14^C*	6-methyluracil-5-sulfate-4,6-^14^C, 35	[81]
	"	", 62	[82]
6	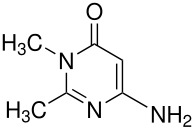 *	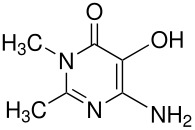 , -	[83]

**Table 8:** Oxidation of quinolones

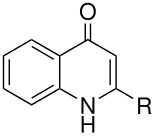			
**# of carbons**	**Quinolone**	**Product(s) and yields (%)**	**Ref.**

9	4-quinolone* (R = H)	3-hydroxy-4-quinolone, 23; 4-quinolone-3-sulfate, 10-15	[6]
10	kynurenic acid* (R = CO_2_H)	3-hydroxykynurenic acid (3-hydroxy-4-quinolone-2-carboxylic acid), 81	[6]
	2-methyl-4-quinolone*	3-hydroxy-2-methyl-4-quinolone, 27; 2-methyl-4-quinolone-3-sulfate, 12	[6]
15	2-phenyl-4-quinolone*	3-hydroxy-2-phenyl-4-quinolone, 40; 2-phenyl-4-quinolone-3-sulfate, 52	[6]
